# Finding Potent Sirt Inhibitor in Coffee: Isolation, Confirmation and Synthesis of Javamide-II (*N*-Caffeoyltryptophan) as Sirt1/2 Inhibitor

**DOI:** 10.1371/journal.pone.0150392

**Published:** 2016-03-17

**Authors:** Jae B. Park

**Affiliations:** Diet, Genomics, and Immunology Laboratory, Bldg. 307C, Rm. 131, BHNRC, ARS, USDA, Beltsville, MD, 20705, United States of America; University of East Anglia, UNITED KINGDOM

## Abstract

Recent studies suggest that Sirt inhibition may have beneficial effects on several human diseases such as neurodegenerative diseases and cancer. Coffee is one of most popular beverages with several positive health effects. Therefore, in this paper, potential Sirt inhibitors were screened using coffee extract. First, HPLC was utilized to fractionate coffee extract, then screened using a Sirt1/2 inhibition assay. The screening led to the isolation of a potent Sirt1/2 inhibitor, whose structure was determined as javamide-II (*N*-caffeoyltryptophan) by NMR. For confirmation, the amide was chemically synthesized and its capacity of inhibiting Sirt1/2 was also compared with the isolated amide. Javamide-II inhibited Sirt2 (IC_50_; 8.7μM) better than Sirt1(IC_50_; 34μM). Since javamide-II is a stronger inhibitor for Sirt2 than Sirt1. The kinetic study was performed against Sirt2. The amide exhibited noncompetitive Sirt2 inhibition against the NAD^+^ (K_i_ = 9.8 μM) and showed competitive inhibition against the peptide substrate (K_i_ = 5.3 μM). Also, a docking simulation showed stronger binding pose of javamide-II to Sirt2 than AGK2. In cellular levels, javamide-II was able to increase the acetylation of total lysine, cortactin and histone H3 in neuronal NG108-15 cells. In the same cells, the amide also increased the acetylation of lysine (K382) in p53, but not (K305). This study suggests that Javamide-II found in coffee may be a potent Sirt1/2 inhibitor, probably with potential use in some conditions of human diseases.

## Introduction

Coffee is one of most popular beverages worldwide[1]. Several recent studies suggest that coffee consumption may have beneficial health effects on several chronic diseases such as diabetes, liver, cancer, and neurodegenerative diseases [2–5]. Alzheimer disease (AD) is a most common neurodegenerative disorder in individuals over age 65, almost 50% of people over the age of 85 [6–8]. Genetically, AD is very heterogeneous, often associated with critical mutations in several genes such as amyloid beta precursor protein (APP), presenilins, apolipoproteins and ABC transporter [9, 10]. Especially, subsequent APP cleavage by the β- and γ-secretase leads to generating amyloid-β (Aβ) peptides which can aggregate and form amyloid plaques [10, 11]. In fact, the pathologic hallmark for Alzheimer disease is the amyloid plaques with neurofibrillary tangles which comprise hyperphosphorylated tau protein [12, 13]. Interestingly, some recent studies suggested potentially positive effects of Sirt2 inhibitors on the progressive degeneration of neurons, although the underlying mechanisms are still under investigation [14–16]. Besides neurodegenerative disorder, regular consumption of coffee was also reported to be positively associated with reduced incidence of some types of cancers [17–18]. Importantly, sertuin1 inhibitors are often recognized as potential therapeutic agents possibly used in treating cancers, because they are capable of increasing the acetylation of p53 [14]. However, the compounds in coffee have not been thoroughly investigated related to Sirt1/2 inhibition, although there are many bio-active compounds in coffee [19, 20]. Therefore, in this paper, coffee extract were prepared, fractionated by a HPLC method and screened in order to explore Sirt1/2 inhibitors in coffee.

## Materials and Methods

Tryptophan, caffeic acid, dichloromethane and other chemicals, were purchased from Sigma Chemical Co. (St. Louis, MO, USA). The Sirt1, 2 and 3 inhibition assays were performed using human Sirt1, 2 and 3 Direct Fluorescent Screening Assay kits (Cayman Co., MA, USA).

### Coffee sample preparation

Coffee samples were prepared using the equal amounts of roasted coffee beans (*Coffea arabica*) from four different coffee brands (Nestle Co., Folgers Co., Maxwell Co, and Starbuck Co.) to resolve possible variety issues for HPLC analyses. After several preparations of coffee samples, the 5g coffee was finely grinned and extracted for 5min with boiling 100mL water. Then, the samples were centrifuged (7000 rpm) and filtered using Millex syringe filter (Millipore, MA). The filtered samples were ready for High Performance Liquid Chromatography (HPLC) analysis.

### HPLC method and fractionation

For HPLC fractionation, a 150 mm × 2.1 mm i.d., 4 μm, Nova-Pak C18 (Waters, Milford, MA) was used as the stationary phase to separate coffee samples. The samples were separated using a gradient condition; buffer A (50 mM NaH_2_PO_4_, pH 4.3) for 0–5 min, a linear change from buffer A to buffer B (40% acetonitril) for 5–40 min, and buffer B for 10 min at the flow rate of 1 mL/min. The samples were injected by an auto-sampler into Alliance 2690 HPLC system (Waters, Milford, MA, USA), and were monitored by CoulArray electrochemical detector with four electrode channels (ESA, Chelmsford, MA, USA). Using the HPLC method, 50 factions were collected at one min interval and each fraction was monitored using the electrochemical detector. The fractions were individually heat-dried and dissolved in 10% ethanol (0.2 mL).

### Sirt1, 2 and 3 inhibition Assay

The Sirt1, 2 and 3 inhibition assays were performed using human Sirt1, 2 and 3 Direct Fluorescent Screening Assay kits (Cayman Co., MA, USA), according to the manufacturer’s manual. The kits provide a convenient fluorescence-based method for screening Sirt1/2/3 inhibitors. The assay was performed in a 96-well microplate. First, human recombinant Sirt1, 2 or 3 is incubated with potential inhibitors. Then, the fluorescent substrate and its co-substrate NAD^+^ were added. The deacetylation by the Sirt2 sensitizes the substrate which releases a fluorescent product with the treatment of developer. Also, the control reactions were performed without Sirt to check fluorescence or quenching effects of the compound/lysate. The fluorophore can be easily analyzed using an excitation wavelength of 350 nm and an emission wavelength of 450 nm. For kinetic study, this assay was performed by diluting NAD+ to the indicated concentrations.

### HPLC purification of Sirt1/2 inhibiting compounds

The fraction with the highest activity was selected, heat-dried and dissolved in 10% ethanol. Then, the sample was injected again into HPLC and separated using the HPLC method used above. The peaks were separately isolated and screened again using the Sirt2 inhibition assay. The peak with the highest activity was saved for NMR analysis.

### NMR analysis of javamide-II

For NMR experiments, the sample was prepared by dissolving javamide-II (20 mg) in d6-DMSO (0.75 mL). ^1^H and ^13^C spectra were acquired at ambient temperature on the JEOL BCX-400 NMR spectrometer operating 400 MHz for ^1^H and 100 MHz for ^13^C.

### Chemical synthesis and NMR confirmation of javamide-II

The synthesis of javamide-II was performed as previously described [21]. Briefly, caffeic acid was dissolved in dimethyl sulfoxide (DMSO) and converted to the symmetrical anhydride with 1,3-diisopropylcarbodiimide (DIC). Tryptophan was first modified with phenylpropanol and added to the reaction mixture. The reaction mixture was incubated at room temperatures with a gentle stirring for 12 hr. The synthesized products were recovered, the phenylpropanol moiety was removed under an alkaline condition, and the final product was purified by HPLC as described previously [21]. The chemical structure was verified using HPLC and NMR spectroscopic methods.

### Kinetic analysis

Kinetic analysis of the Sirt2 inhibition was performed, and K_i_ value was determined using Lineweaver-Burk plot. Data points in all figures represent the mean ± SD (n = 3).

### Molecular Docking

Molecular docking study was performed using an algorithm-based docking program ICM-pro (MolSoft, San Diego, CA). The proposed binding to the active pocket of the Sirt enzymes was determined as the best ranked scoring function, representing the conformational structures with the most favorable free binding energy (ΔG_binding_).

### Cell Culture

Neuroblastoma×glioma (NG108-15) cells were grown in DMEM supplemented with 10% (v/v) heat-inactivated fetal bovine serum at 37°C in a humidified atmosphere of 5% CO_2_.

### Western blot

For the Western blot, the extracts were prepared using NG108-15 cells treated with various concentrations of javamide-II (0, 5, 10, 20) for 18 hr. The amounts of protein in the extracts were determined using Bio-Rad protein assay kit (Hercules, CA, USA) and an equal amount protein was used per samples for the blot. The Western blots for total acetylated lysine and alpha-tubulin were performed using Novex 4–12% Tris-Glycine Mini Gel and XCell II^™^ Blot Module kit (LifeTechnologies, Cambridge, MA, USA), acetylated-cortactin antibody (EMD Millipore, MA, USA) and acetylated-lysine, acetylated-histoneH4, acetylated-histoneH4(K56), acetylated-p53, and alpha-tubulin antibodies (Cell Signal, Danvers, MA, USA).

### Assays for histone H3, p53 and α-Tubulin acetylation

The quantification of histone H3 acetylation was performed using a histone H3 total acetylation detection kit (Abcam, Cambridge, MA, USA). The extracts were prepared using NG-108 cells treated with various concentrations of javamide-II (0, 1, 5, 10, 20) for 24 hr, according to the kit’s manual. The extracts were used immediately or stored at–80°C for later use. The equal amounts of the protein determined by Bio-Rad Protein Assay Kit (Hercules, CA, USA) were used for the determination of histone H3 acetylation. Similarly, the measurement of p53 and α-tubulin acetylation was also performed using p53 acetyl K382 Human ELISA kit and p53 Acetyl K305 Human ELISA Kit (Abcam, Cambridge, MA, USA), and PathScan^®^ acetylated-alpha-tubulin Sandwich ELISA kit (Cell Signal, Danvers, MA, USA) as described in the manufacturers' manuals.

### Statistical Analysis

Treatment effects on the parameters measured were compared by analyzing the means for differences using One-way ANOVA with Holm-Sidak method. Differences were considered to be significant when p< 0.05. Data points represent the mean ± SD of three or more samples.

## Results

### Coffee extraction and HPLC fractionation

**Fig 1:**
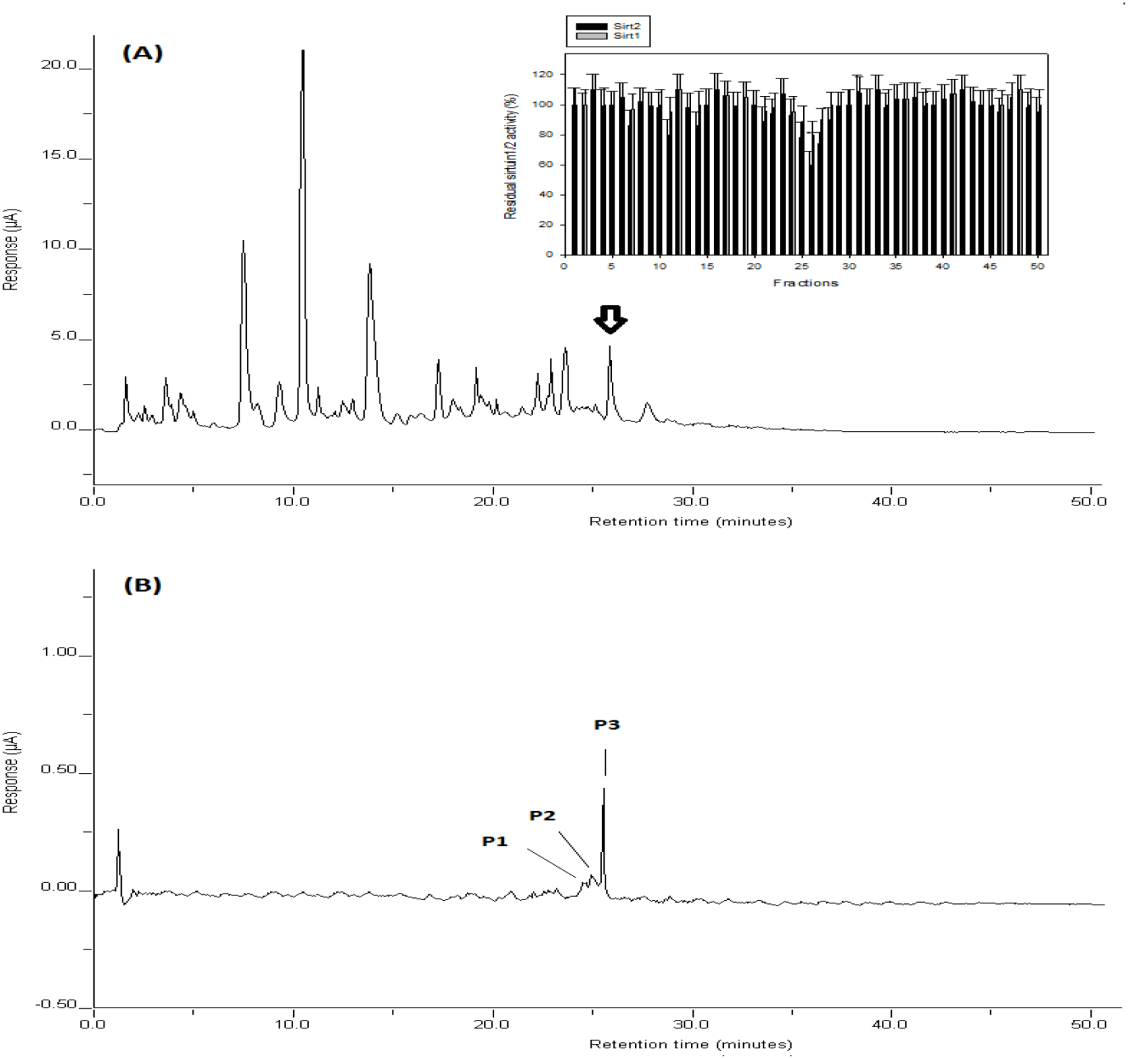
HPLC chromatograms of coffee samples. Coffee samples were prepared and fractionated as described in “Materials and Methods”. The peaks were detected using an electrochemical detector. (A) Using HPLC fractions, Sirt1/2 inhibition assay (Insert) was performed as described in “Materials and Methods”. The marks (*) indicate statistical significance (P<0.05). (B) HPLC isolation of Sirt2 inhibiting compounds. Data are presented as mean ± SD (n = 6). P value was calculated using one-way ANOVA with Holm-Sidak method and the marks (*) indicate statistical significance (P<0.05).

In this study, coffee samples were prepared using roasted coffee from four different coffee brands to resolve possible variety issues for the high-performance liquid chromatography (HPLC) analyses. For an optimal HPLC fractionation, a new HPLC method was developed and utilized to fractionate the coffee sample as described in “Materials and Methods”. Several different buffer conditions were tested to improve the resolution of the coffee compounds with a reliable detection, and then a gradient condition was selected for optimizing separation, detection and sample collection as described in "Materials and Methods". The chromatograms of the coffee sample were shown in Fig 1A. Total HPLC running time was about 50 min for the assay. Three dominant peaks were detected at the retention times (RT) of 7.7, 12.1, 14.5 min, and some minor peaks at 17.5, 19.4, 22.2, 23.8, 24.2, 26.1 and 28.2 min (Fig 1A).

### Sirt1/2 inhibition assay using HPLC fractions

The fractions were individually heat-dried, and then dissolved in the buffer A for Sirt1/2 inhibition assay as described in "Materials and Methods". As shown in Fig 1A, the highest inhibition of Sirt1/2 was detected in the fraction (F26), although some fractions (F7, F11 and F14) were also found to have some extent of Sirt1/2 inhibiting activity. Since the fraction (F26) contained the highest inhibition activity, the fraction was heat-dried and the residue was prepared in the buffer A. Then, the sample was re-injected into HPLC, and separated using the gradient condition as described in "Materials and Methods". As shown in Fig 1B, one major and two minor peaks were detected in the fraction. The peaks were individually collected, the samples were separately heat-dried, prepared and tested. A major peak (RT of 26 min) was found to have most significant Sirt1/2 inhibiting activity (Table 1). Meanwhile, two minor peaks (RTs of 24 and 25 min) showed nearly no activity. These data indicated that the major peak may have most of Sirt2 inhibiting activity in the fraction (F26).

**Table 1:** Sirt1/2 inhibition by HPLC peaks. Sirt1/2 inhibition activity was measured as described in “Materials and Methods”. Data are presented as mean ± SD (n = 6). ND; not detected.

Peaks	Sirt1 inhibition (%)	Sirt2 inhibition (%)
Peak1	ND	2±2.1%
Peak2	ND	4±3.0%
Peak3	19±8.9%	35±8.9%

### NMR analysis of the Sirt1/2 inhibiting compound

**Fig 2:**
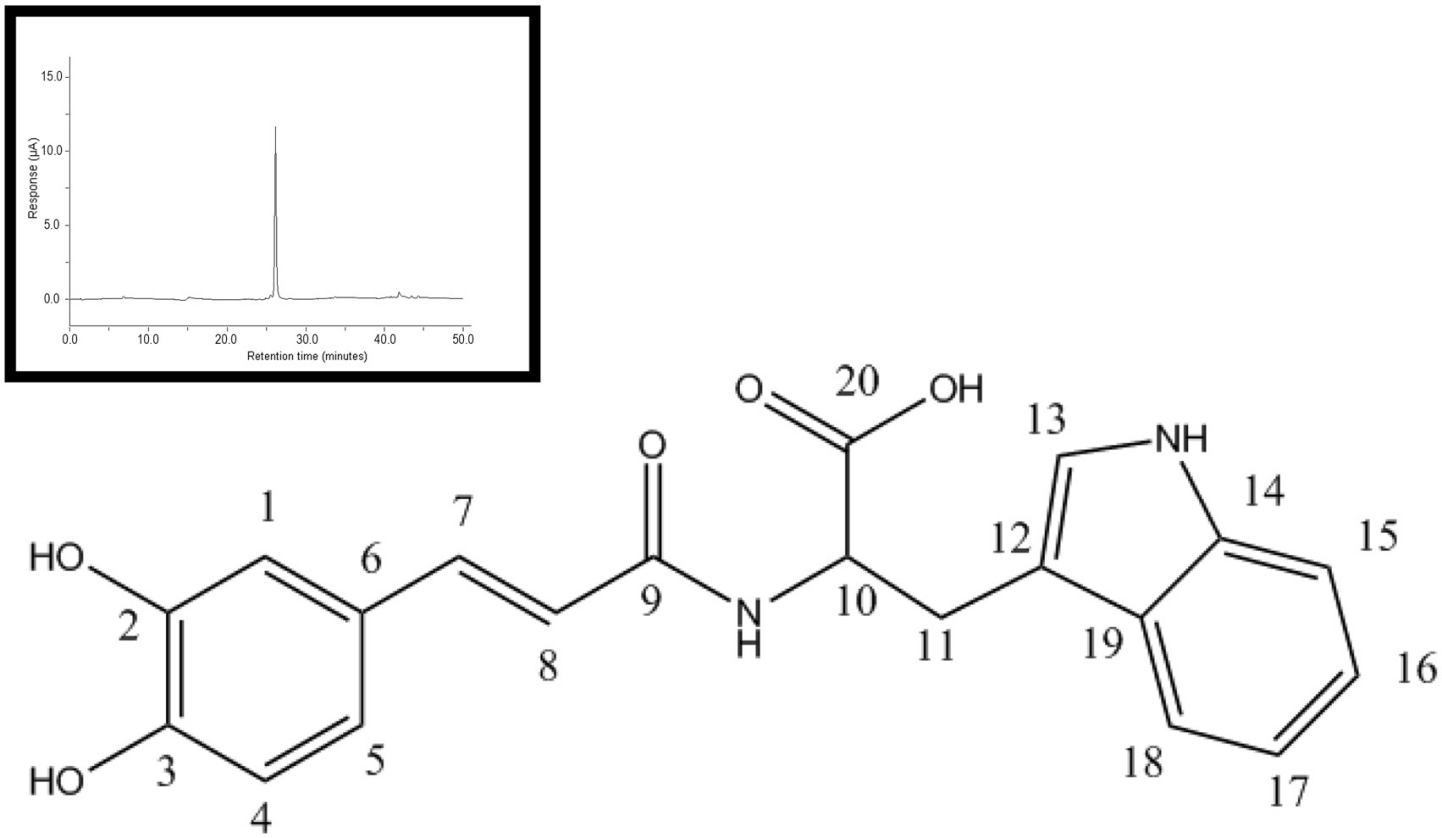
Chemical structure of javamide-II. The chemical synthesis of javamide-II was performed as described in “Materials and Methods”. The chemical structure was verified using HPLC (an insert) and NMR spectroscopic methods.

To determine the identity of the peak, NMR analyses were performed as described in "Materials and Methods". For NMR experiments, the sample was prepared by dissolving javamide-II (20 mg) in d6-DMSO (0.75 mL). ^1^H and ^13^C spectra were acquired at ambient temperature on the JEOL BCX-400 NMR spectrometer operating 400 MHz for ^1^H and 100 MHz for ^13^C. Chemical shifts were referenced to DMSO (2.50 ppm for ^1^H, 39.5 ppm for ^13^C). The NMR data were following: 1H NMR (d6-DMSO, 400 MHz) 7.33 ((1H, d, *J*) 15.6 Hz, H-7), 7.20 ((1H, s, H-13), 7.08 ((1H, t, *J*) 7.3 Hz, H-16), 6.68 ((1H, dd, *J*) 8.2, 1.4 Hz, H-5), 6.41 ((1H, d, *J*) 15.6 Hz, H-8), 7.58 ((1H, d, *J*) 8.2 Hz, H-18), 6.99 ((1H, t, *J*) 7.3 Hz, H-17), 6.81 ((1H, d, *J*) 8.7 Hz, H-4), 7.05 (1H, s, H-1), 7.35 ((1H, d, *J*) 7.8 Hz, H-15), 4.71 ((1H, dt, *J*) 6.0, 6.9 Hz, H-10), 2.94 ((1H, t, *J*) 7.3 Hz, H-11), 8.19 ((1H, t, *J*) 5.5 Hz, H-a), 10.79 (1H, s, H-b); 13C NMR (d6-DMSO, 100 MHz) 174.7 (C, C-20), 166.7 (C, C-9), 147.5 (C, C-3), 145.7 (C, C-2), 141.5 (C, C-7), 136.4 (C, C-14), 127.4 (C, C-19), 126.9 (C, C-6), 122.8 (C, C-13), 121.1 (C, C-16), 120.2 (C, C-5), 118.9 (C, C-8), 119.5 (C, C-17), 118.4 (C, C-18), 117.0 (C, C-4), 115.0 (C, C-1), 112.1 (C, C-12), 111.5 (C, C-15), 58.7 (C, C-10), 26.2 (C, C-11). Based on the NMR data, the structure of the HPLC purified compound with Sirt2 inhibiting activity was determined as being (E)-(3-(3,4-dihydroxyphenyl)acryloyl)tryptophan (*N*-caffeoyltryptophan), and the compound was named as javamide-II (Fig 2).

### Chemical synthesis and activity confirmation of javamide-II

To further demonstrate that javamide-II is the compound able to inhibit Sirt1/2, the synthesis of javamide-II was performed as previously described [21]. Briefly, caffeic acid was dissolved in dimethyl sulfoxide (DMSO) and converted to the symmetrical anhydride with 1,3-diisopropylcarbodiimide (DIC). Tryptophan was first modified with phenylpropanol and added to the reaction mixture. The reaction mixture was incubated at room temperatures with a gentle stirring for 12 hr. The synthesized products were recovered and the final product was purified by HPLC as described previously [21]. The chemical structure was also verified using HPLC and NMR spectroscopic methods. The NMR data were the same as the data of the isolated javamide-II (Data not shown here). Using isolated and synthesized javamide-II, their capacity of inhibiting Sirt1/2 was compared at several different concentrations. They were found to have very similar inhibition pattern (Table 2), suggesting that javamide-II isolated from coffee was the compound with Sirt1/2 inhibition activity.

**Table 2:** Comparison of Sirt1/2 inhibition of isolated and synthesized javamide-II. Sirt1/2 inhibition activity was measured as described in “Materials and Methods”. Data are presented as mean ± SD (n = 6). The data in the parentheses are Sirt1 inhibition.

Concentrations (μM)	Residual Sirt1/2 activity	
	Isolated	Synthesized
0	100±11.9 (100±9.7)%	100±10.1 (100±9.9)%
1	81±9.9 (91±9.5)%	80±10.9 (90±9.7)%
5	61±9.2 (82±9.1)%	60±9.9 (81±8.5)%
10	41±8.8 (76±8.5)%	41±9.0 (76±9.1)%
20	31±8.2 (65±9.5)%	31±8.7 (64±9.5)%

### The specificity of javamide-2 to Sirt1, 2 and 3

The specificity of inhibitors is often regarded as a very critical factor in determining which inhibitor may be appropriate in treating disease conditions. In fact, Sirt proteins are found in specific cellular compartment; for instance, Sirt1, 6 and 7 are found in the nucleus, Sirt2, and often Sirt1 are found in the cytoplasm, and Sirt3, 4 and 5 are found in the mitochondria in human cells. Since javamide-II showed strong Sirt2 inhibition activity, the efficacy of the amide was also investigated related to srituin1 (nucleus) and 3 (Mitochondria) was investigated. As shown in Table 3, the amide inhibited Sirt2 stronger than Sirt1 and 3, especially Sirt3. Although javamide-II inhibited Sirt2 better than Sirt1, the specificity to the two enzyme was very moderate and javamide-II could inhibit Sirt1 effectively.

**Table 3:** Specificity of javamide-II to Sirt1, 2 and 3 enzymes. Inhibition ratio average is expressed as tested Sirt/Sirt2. Data are presented as mean ± SD (n = 6).

(μM)	Inhibition (%)		
	Sirt1	Sirt2	Sirt3
0	0	0	0
0.1	5.2±3.4%	13±4.9%	1.5±1.7%
5.0	18±4.2%	39±5.9%	5.7±2.7%
10	24±5.8%	59±7.0%	8.0±3.7%
20	35±5.9%	69±8.7%	11±4.7%
40	59±6.1%	87±9.1%	28±6.1%
60	75±9.2%	98±9.7%	50±8.1%
**Ratio Average**	0.54	1	0.22

### Kinetic study of Sirt inhibition

**Fig 3:**
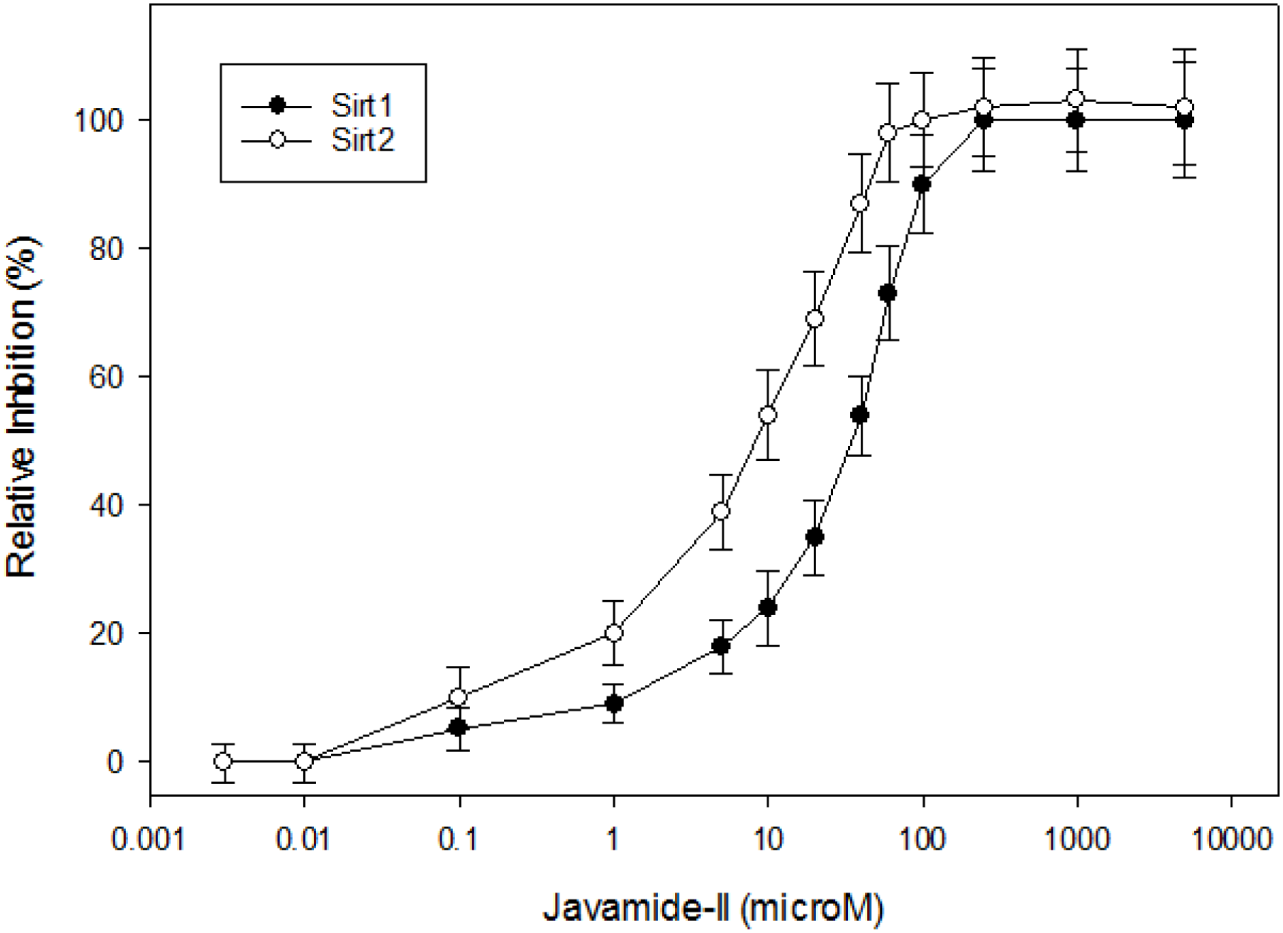
IC_50_ values of javamide-II against Sirt1 and 2. IC_50_ was determined using measuring Sirt1 and 2 inhibition at the range of javamide-II between 0.003 and 5000 μM. Data points in all figures represent the mean ± SD (n = 3).

**Fig 4:**
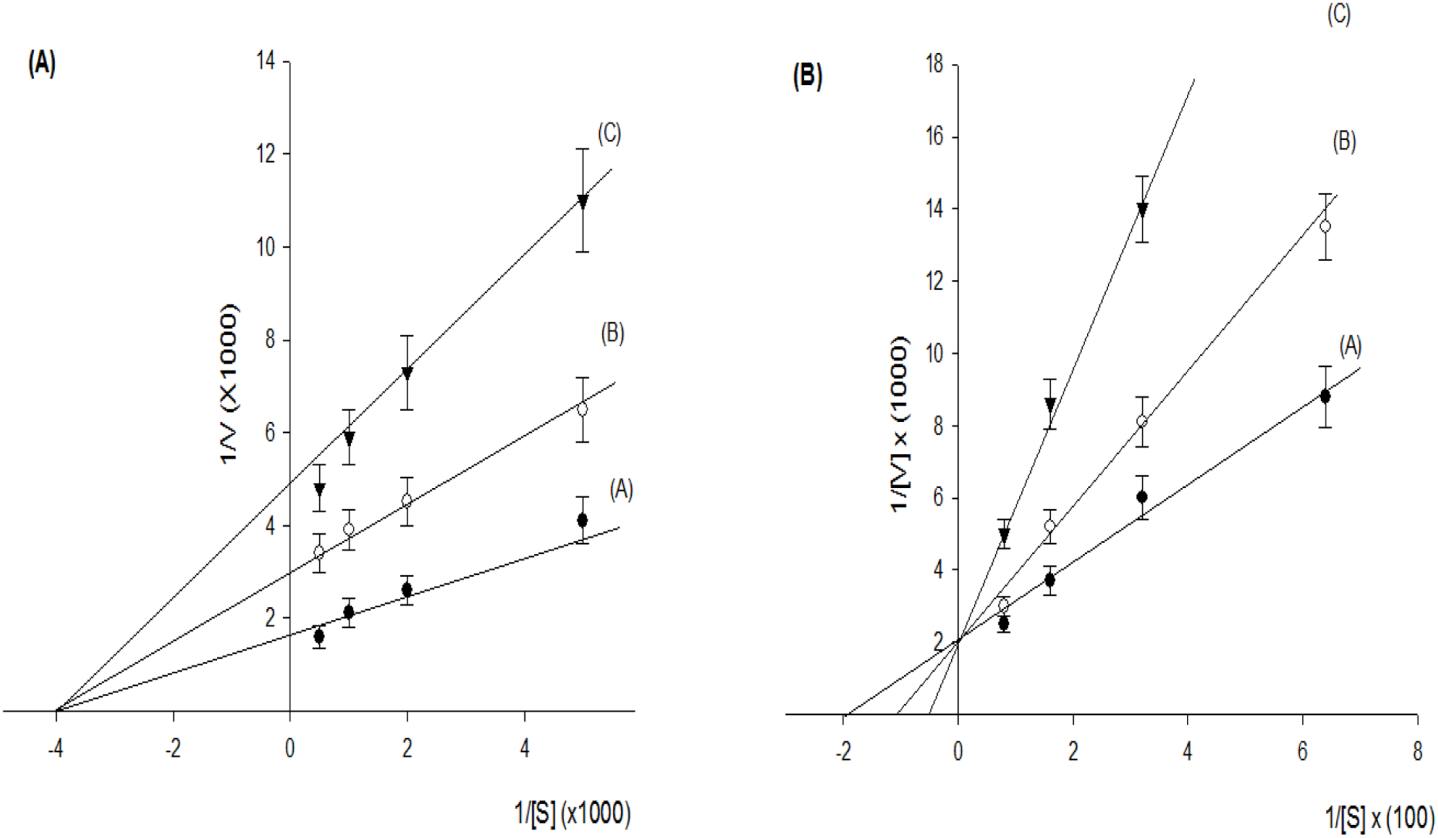
Kinetic study of Sirt2 inhibition by javamide-II. (A) K_i_ value was determined using Lineweaver-Burk plot. A is a control, and B and C with 10 and 20 μM javamide-II, respectively. The inhibition activity was measured with NAD^+^ at 200, 500, 1000, and 2000 μM. (B) K_i_ value was determined using Lineweaver-Burk plot. A is a control, and B and C with 5 and 10 μM javamide-II, respectively. The inhibition activity was measured with peptide substrate at 15.6, 31.2, 62.5, and 125 μM. Data points in all figures represent the mean ± SD (n = 3).

Javamide-II inhibited Sirt2 (IC_50_ = 8.7 μM) stronger than Sirt1 (IC_50_ = 34 μM) (Fig 3), which is comparable to AGK [22] (Sirt2 IC_50_ = 4.3 μM) and EX527 [23] (Sirt2 IC_50_ = 5.9 μM) performed in our laboratory. Therefore, kinetic experiments were performed to determine the K_i_ values of javamide-II against Sirt2. As shown in Fig 4A, the amide inhibited Sirt2 noncompetitively with respect to NAD^+^, and the K_i_ value was approximately 9.8 μM. Additionally, the amide showed competitive inhibition against the peptide substrate with the K_i_ value of 5.3 μM (Fig 4B). These data indicate that javamide-II found in coffee may be a potent Sirt2 inhibitor, which may inhibit Sirt2 at relatively low concentrations.

### Molecular docking

**Fig 5:**
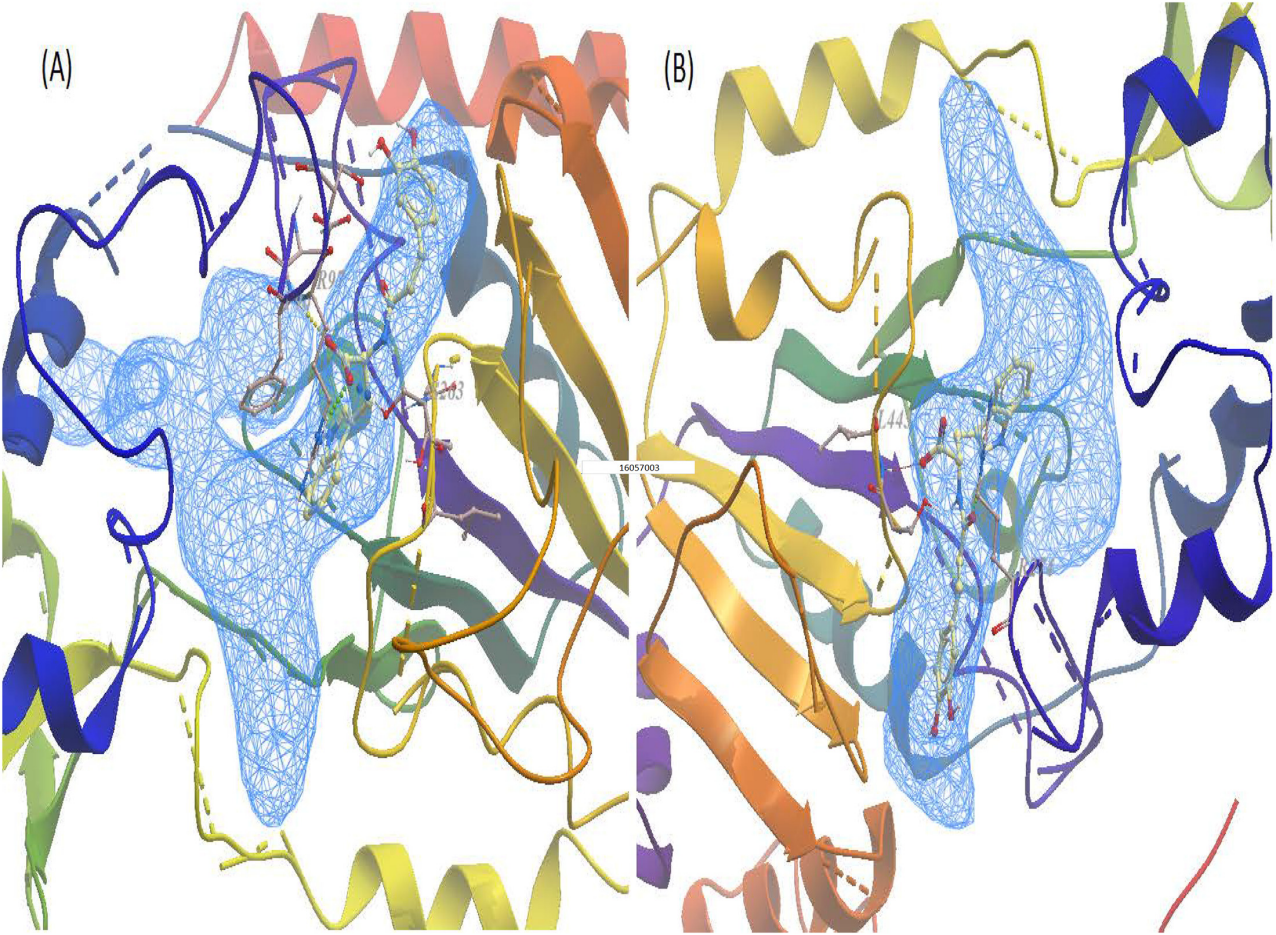
Javamide-II docked into Sirt1/2. (A) Best-ranked javamide-II to Sirt2 enzyme were presented (4RMJ, a putative pocket) and three potential hydrogen bonds were dotted in yellow (F96), green (R97) and blue (S263). (B) Best-ranked javamide-II to Sirt1 enzyme were presented (4I5I, a putative pocket) and two potential hydrogen bonds were dotted in red (L443) and green (S274).

Since javamide-II demonstrated strong inhibitory activity against Sirt2 better than Sirt1 and 3, a qualitative molecular docking were first performed with javamide-II against 8 human Sirt2 complexes in order to support the observed Sirt2 inhibition (Table 4). This docking study was performed using an algorithm-based docking program ICM-pro as described in "Material and Methods". The docking experiments were performed using javamide-II and AGK2 (Sirt2 inhibitor) on the available experimental co-crystallized Sirt2 complexes for (8 complexes, Table 4) [24–27]. Briefly, the Sirt2 complex was divided into ligand and protein, then the ligands (javamide-II and AGK2) were docked into the protein complexes. The resulting lowest energy and other scoring values were compared (Table 4). Overall, javamide-II exhibited better scores than AGK2. In Fig 5A, the lowest energy pose of javamide-II was selected as the likely binding modes for the amide found in Sirt2 protein (PDB_ID; 4RMJ) and potential hydrogen bonds were depicted in green and blue. In addition, the similar docking experiments were performed using javamide-II and EX527 on the available experimental co-crystallized Sirt1 complexes (Table 5) [28–30], and the lowest energy pose of javamide-II was selected as the likely binding modes for the amide found in Sirt1 protein (PDB_ID; 4I5I) and potential hydrogen bonds were depicted in green and red (Fig 5B).

**Table 4:** Sirt2 docking scores of javamide-II and AGK2. Docking scores and energy values were obtained using a docking program ICM-pro as described in "Material and Methods".

Sirt2 (PDB_ID)	Javamide-II		AGK2	
	Score	ΔE (Kcal/mol)	Score	ΔE (Kcal/mol)
1J8F (24)	-28.4	-32.5	-29.7	-19.2
3ZGO (25)	-27.4	-31.5	-21.4	-12.5
3ZGV (25)	-32.5	-45.3	-29.1	-30.1
4L3O (26)	-33.6	-40.6	-19.5	-15.6
4RMG (27)	-21.3	-37.8	-20.9	-25.4
4RMH (27)	-23.6	-42.3	-34.5	-36.2
4RMI (27)	-29.4	-40.4	-32.5	-29.6
4RMJ(27)	-47.7	-58.6	-25.1	-27.8
**Average**	-32.5	-45.5	-26.6	-24.6

**Table 5:** Sirt1 docking scores of javamide-II and EX527. Docking scores and energy values were obtained using a docking program ICM-pro as described in "Material and Methods".

Sirt1 (PDB_ID)	Javamide-II		EX527	
	Score	ΔE (Kcal/mol)	Score	ΔE (Kcal/mol)
4IF6	-34.4	-43.5	-16.7	-37.1
4IG9 (28)	-26.4	-31.5	-15.7	-23.5
4KXQ (28)	-27.7	-45.3	-11.9	-37.4
4I5I (29)	-45.4	-42.7	-41.7	-35.4
4ZZH (30)	-33.6	-37.8	-11.5	-25.3
**Average**	-33.5	-40.2	-19.5	-31.7

### Western blot for total acetylated lysine

**Fig 6:**
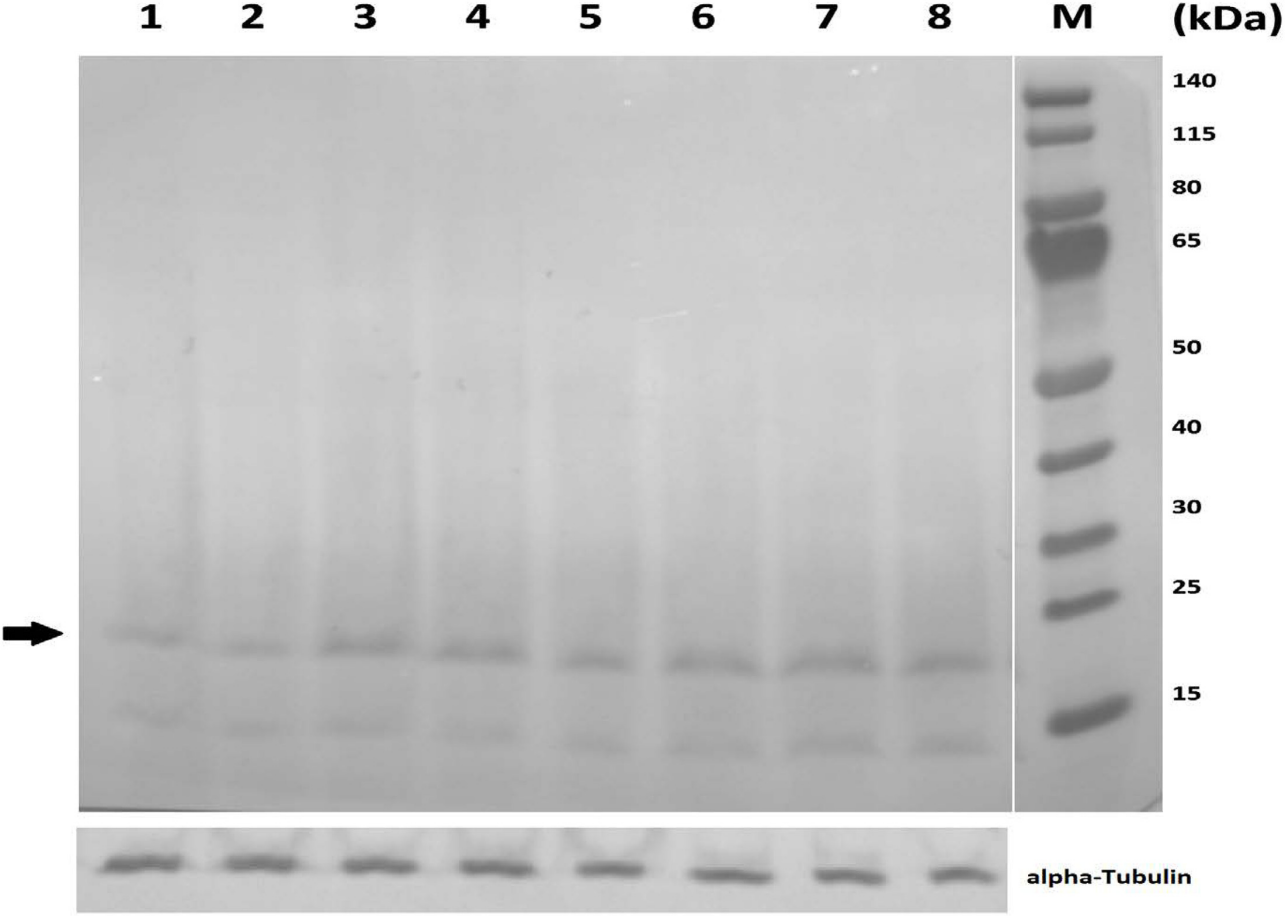
Effects of javamide-II on total acetylated lysine. The nuclear extracts were prepared using the NG108-15 cells treated with various concentrations of javamide-II (0, 5, 10, 20 μM) for 18 hr. Control (1 and 2 lanes), 5 μM (3 and 4 lanes), 10 μM (5 and 6 lanes), 20 μM (7 and 8 lanes) and M (protein molecular weight markers). Tubulin blot was provided to show the levels of proteins in the extract used in the blot.

Because javamide-II was a stronger inhibitor for Sirt2 than Sirt1, that could be more relevant to neurodegenerative diseases. Therefore, the extracts were prepared using the neuroblastoma×glioma NG108-15 cells treated with various concentrations of javamide-II (0, 5, 10, 20 μM). As shown in Fig 6, the total acetylation was increased with the treatment of javamide-II, suggesting that the amide likely inhibited the deacetylation process in the cells. Based on the protein marker, the band with the arrow mark could be an acetylated histone H3 of which the signal showed about 2-fold increase in the blot. Therefore, we investigated potential effects of javamide-II on the acetylation levels in histone H3 protein

### Cellular effects of javamide-II on the acetylation of histone H3

**Fig 7:**
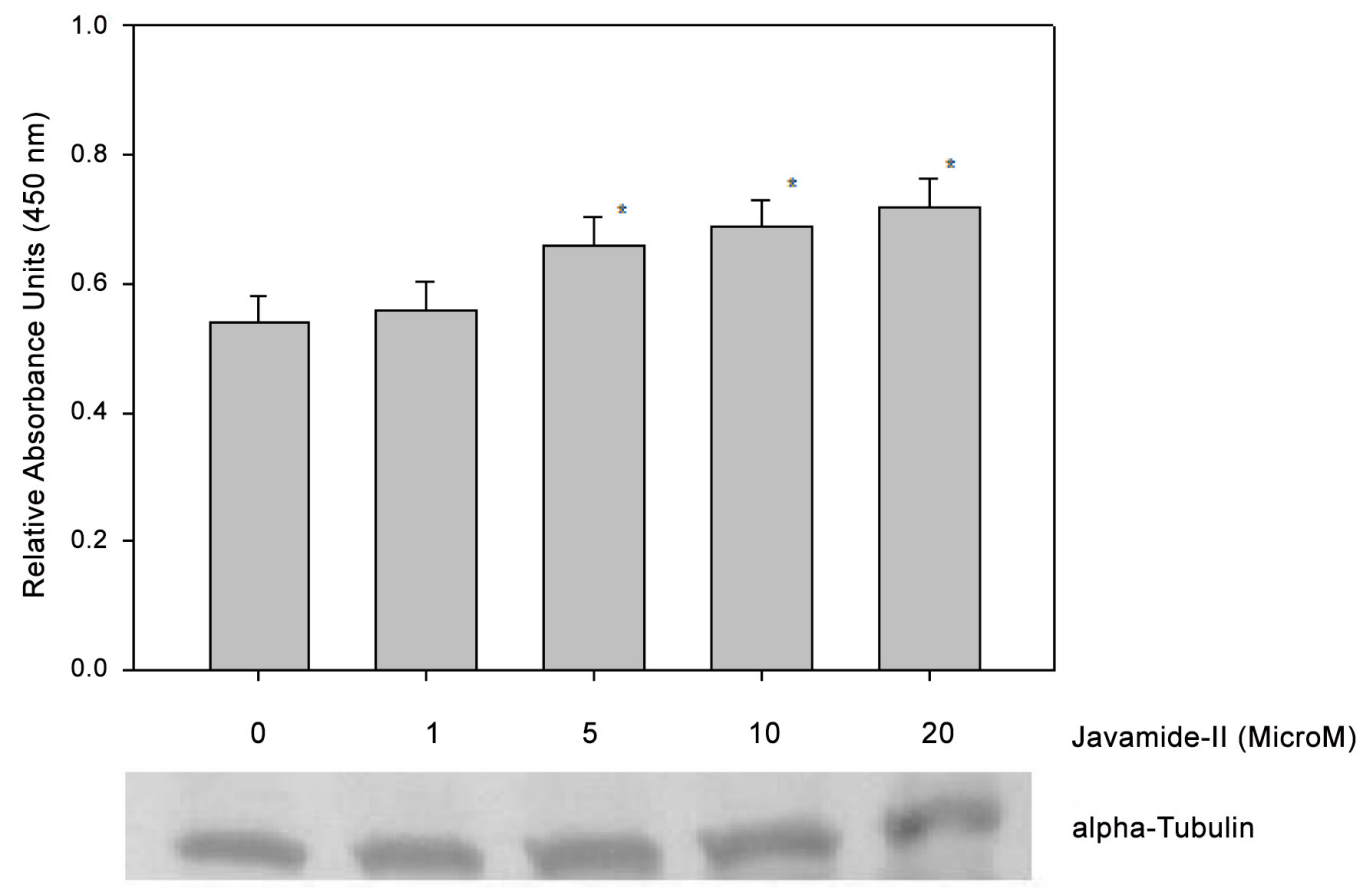
Effects of javamide-II on histone H3 acetylation. The histone extracts were prepared using the NG108-15 cells treated with various concentrations of javamide-II (0, 1, 5, 10, 20 μM) for 18 hr, according to the kit’s manual. Data are presented as mean ± SD (n = 6). P value was calculated using one-way ANOVA with Holm-Sidak method and the marks (*) indicate statistical significance (P<0.05). Tubulin blot was provided to show the levels of proteins in the extract.

Because javamide-II was identified as a better inhibitor for Sirt2 than Sirt1 and because histone H3 is a well-known substrate for Sirt2 [31], we investigated the potential effects of javamide-II on the acetylation of histone H3. As shown in Fig 7, the acetylation was increased with the treatment of javamide-II, suggesting that the amide did inhibit the deacetylation in the NG108-15 cells. These data let us to investigate other histone protein (e.g., histone H4) which is also considered as a substrate for Sirt2. For this experiment, the histone H4 acetylation assay ELISA kit (Abcam, Cambridge, MA, USA) was used to investigate potential effects of javamide-II on histone H4 in the cell. However, we could not detect a significant acetylation increase in histone H4 (Data not shown here).

### Effects of javamide-II on the acetylation of alpha-tubulin

Like histone H3, α-tubulin is a known substrate for Sirt2. Therefore, we did investigate the potential effects of javamide-II on the acetylation of α-tubulin in NG108-15 cells. However, the α-tubulin acetylation was not increased significantly with the treatment of javamide-II in the NG108-15 cells (Data not shown here).

### Effects of javamide-II on p53 acetylation

**Fig 8:**
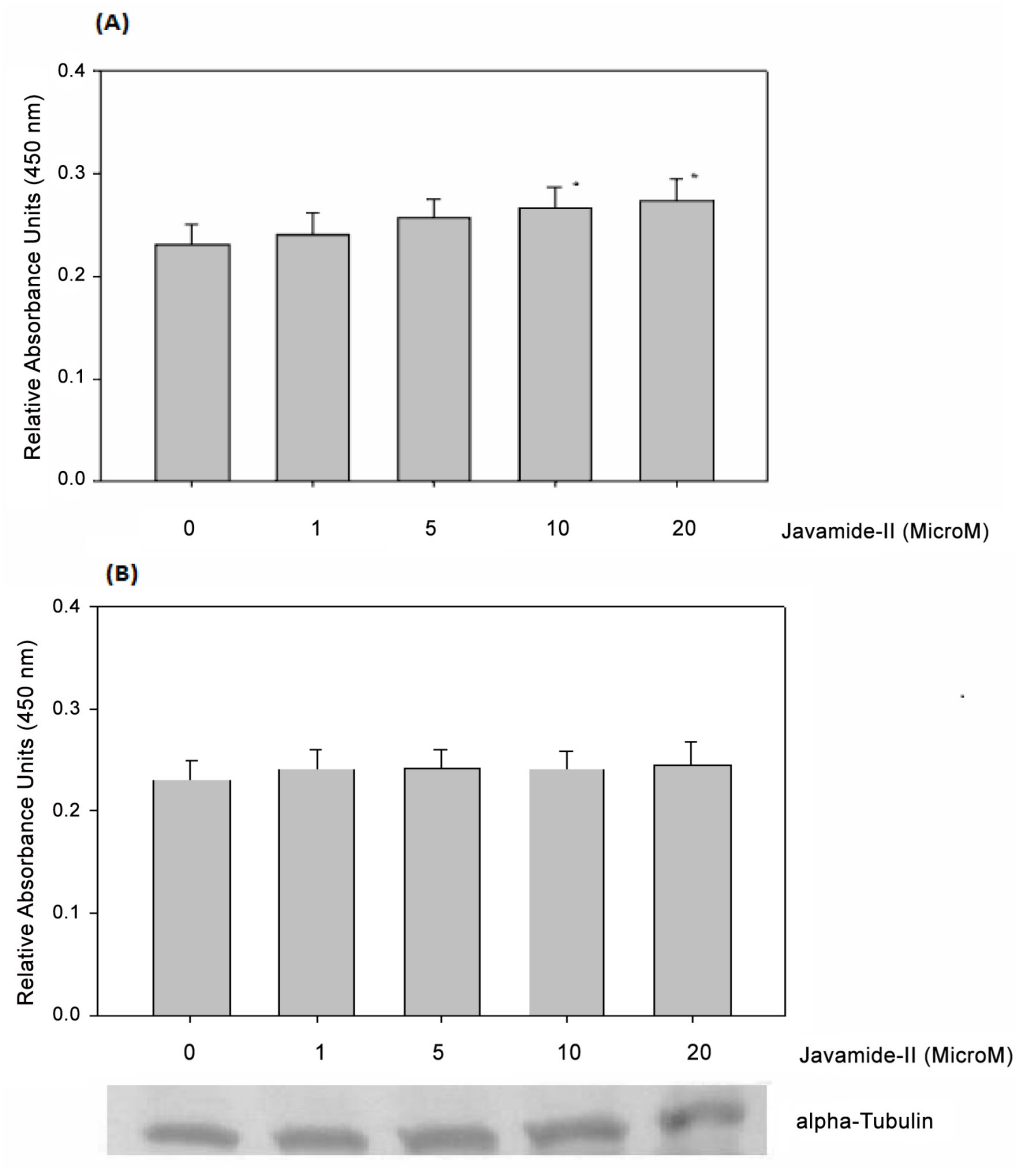
Effects of javamide-II on p53 acetylation. (A) p53 acetylation at K382. The nuclear extracts were prepared using the NG108-15 cells treated with various concentrations of javamide-II (0, 1, 5, 10, 20 μM) for 18 hr, according to the kit’s manual. (B) p53 acetylation at K305. The nuclear extracts were prepared as described in (A). Data are presented as mean ± SD (n = 6). P value was calculated using one-way ANOVA with Holm-Sidak method and the marks (*) indicate statistical significance (P<0.05). Tubulin blot was provided to show the levels of proteins in the extract.

Another famous substrate for Sirt1/2 is p53 which is a potent transcription factor involved in cell cycle arrest and apoptosis via activating numerous genes, and its growth suppressive and pro-apoptotic activity could be utilized in fighting cancer cells [32]. Therefore, we investigated the potential effects of javamide-II on the acetylation of p53 in NG108-15 cells. The treatment with javamide-II led to increase in the p53 acetylation, suggesting that the amide did inhibit the deacetylation of p53 in the cells (Fig 8A). In fact, the p53 assay was performed using p53 acetyl K382 human ELISA kit which primarily detects the acetylation of lysine (K382) in human p53. Therefore, we further investigated the acetylation of other sites in the p53 such as the lysine (K305) using a p53 acetyl K305 human ELISA Kit. However, the acetylation of lysine (K305) was little effected with the treatment of javamide-II (Fig 8B). It is likely that javamide-II may inhibit the deacetylation of p53 specifically. Nonetheless, all these data suggest that javamide-II found in coffee may be a potent Sirt1/2 inhibitor, which can inhibit the deacetylation of p53 in the NG108 cells.

### Western blot for histone H3, alpha-tubulin, p53 and cortactin acetylation

**Fig 9:**
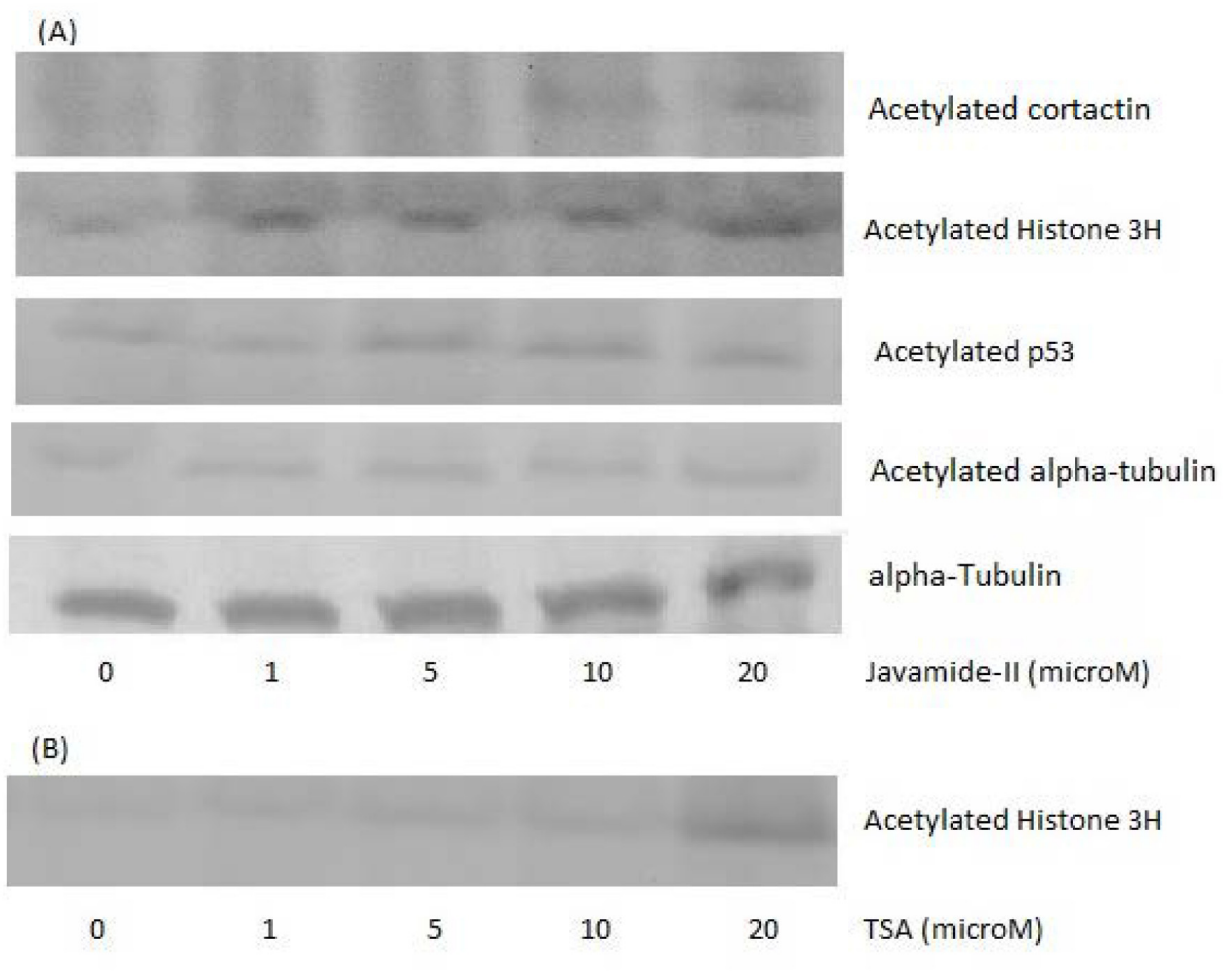
Western blots for histone H3, alpha-tubulin and p53 acetylation. The nuclear extracts were prepared using the NG108-15 cells treated with various concentrations of javamide-II (0, 1, 5, 10, 20 μM) for 18 hr. (A) Western blots for histone H3, p53 and alpha-tubulin acetylation in the cells treated with javamide-II. For the Western blots, the similar amounts of nuclear extract were loaded, determined by protein assay, and also, tubulin blot was provided to show the levels of proteins in the extract used in the blot. (B) Western blot for histone H3 acetylation in the Trichostatin A-treated cells was provided with as a positive control (TSA; 0, 1, 5, 10, 20 μM).

**Fig 10:**
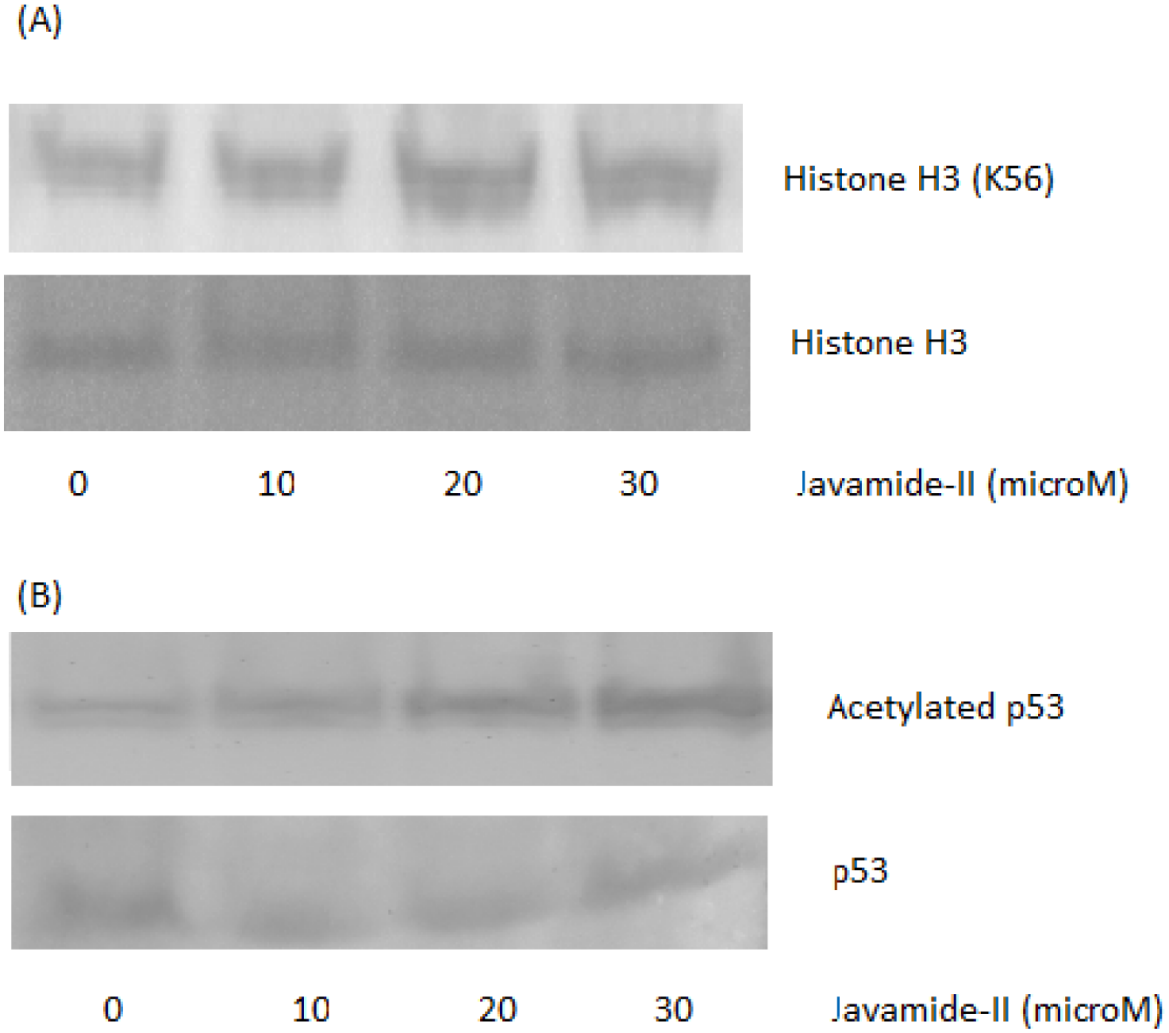
Western blots for histone H3 and p53 acetylation. The nuclear extracts were prepared using the NG108-15 cells treated with various concentrations of javamide-II (0, 10, 20, 30 μM) for 18 hr. For the Western blots, the similar amounts of nuclear extract were loaded, determined by protein assay. (A) Western blots for histone H3 (K56) acetylation in the cells treated with javamide-II. Histone H3 blot was provided to show the levels of proteins in the extract used in the blot. (B) Western blot for p53 acetylation in the cells treated with javamide-II and etoposide (10 μM). p53 blot was provided to show the levels of proteins in the extract used in the blot.

For validating the ELISA data, we also performed Western blots using the extracts prepared using the NG108-15 cells treated with javamide-II (0, 1, 5, 10, 20 μM). As shown in Fig 9, the acetylation of H3 and p53 (K382) was increased with the treatment of javamide-II, demonstrating that ELISA data were clearly in a line with the those of Western blots. However, the acetylation of alpha-tubulin was not increased with the treatment of javamide-II again, also in a line with the ELISA data. Since alpha-tubulin acetylation was not increased significantly, we investigated potential effects of javamide-II on another Sirt2 substrate (cortactin) [33]. The treatment of NG108-15 cells with javamide-II increased its acetylation in a dose-dependent way (Fig 9). These data suggest that the treatment of javamide-II could increase histoneH3, cortactin, and p53 acetylation in NG108-15 cells. Although the acetylation of H3 was increased with the treatment of javamide-II, the detection was carried out using the antibody able to detect histone H3 acetylation in non-site specific manner. Therefore, we further investigated histone H3 acetylation using a site-specific acetyl-histone H3 (K56) antibody, which site is also known to be deacetylated by Sirt1 and 2 [22]. As shown in Fig 10A, the acetylation of histine H3 at the site was increased with the treatment of javamide-II (0, 10, 20, 30 μM). These data suggest that the javamide-II treatment can increase the acetylation of histone H3 specifically at lysine56. Additionally, we also investigated the acetylation of p53 in the presence of etoposide. As shown in Fig 10B, the acetylation of p53 was increased more with the treatment of both javamide-II and etopopside than etoposide alone. Again, all these data indicated that the treatment of javamide-II could increase histoneH3, cortactin, and p53 acetylation in NG108-15 cells.

## Discussion

**Fig 11:**
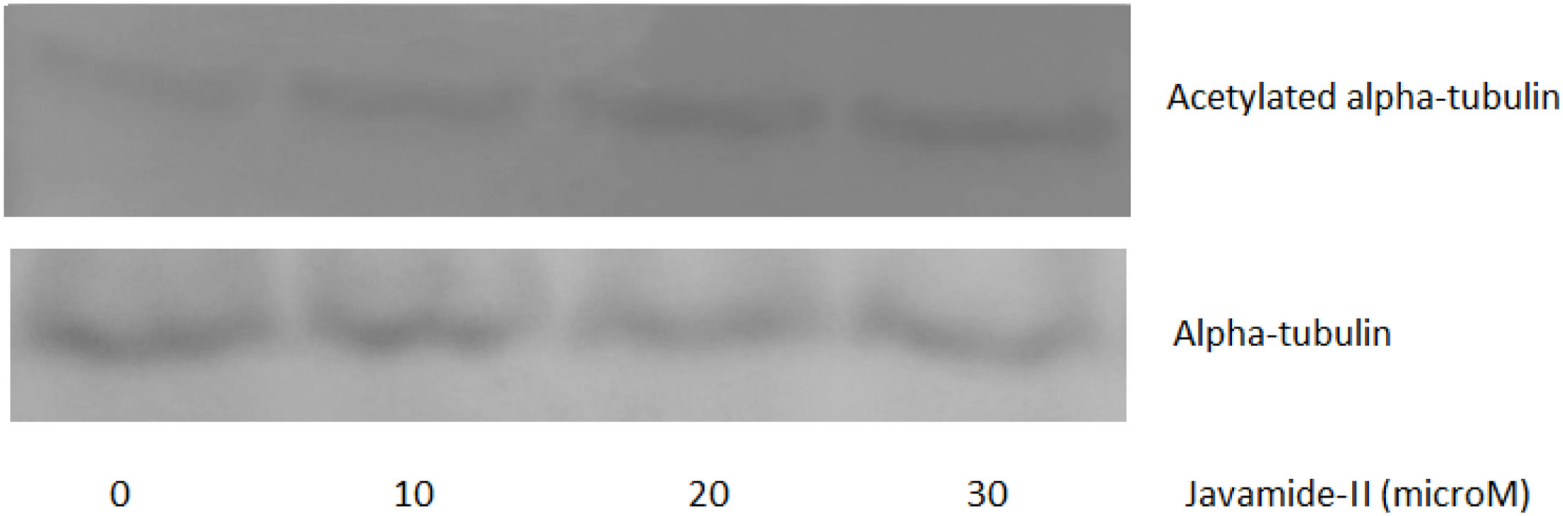
Western blots for alpha-tubulin acetylation in THP-1 cells. The nuclear extracts were prepared using the THP-1 cells treated with various concentrations of javamide-II (0, 10, 20, 30 μM) for 18 hr. Western blots for alpha-tubulin acetylation in the cells treated with javamide-II. Tubulin blot was provided to show the levels of proteins in the extract used in the blot.

The Sirt consists of a family of seven proteins with highly conserved NAD^+^-binding domain (Sirt1–7) [14, 34–43]. Because all Sirt proteins have deacetylase activity [41–44], they can deacetylate internal acetylated lysine residues at their ε-amino groups. This reversible post-translational acetylation modification swiftly manipulates a protein’s activity, thereby regulating critical processes such as metabolism, proliferation and cell death [34–44]. Therefore, the Sirt enzymes have become attractive target molecules in many scientific fields, due to their broad role in metabolism, aging and cancer. Alzheimer disease (AD) is a serious neurodegenerative disorder of which impact is gravely profound in personal, social and financial levels. Therefore, therapeutic agents preventing/treating this disease have been sought relentlessly for years. In this study, javamide-II found in coffee was screened/isolated as a potential Sirt2 inhibitor candidate, possibly utilized for human diseases including Alzheimer's disease and other diseases. However, during the course of the study, we found that the javamide-II also could exert other potential important activity such as increased acetylation of p53 protein probably via inhibiting Sirt1/2. Also, our preliminary data suggested that javamide-II (higher than 30 μM) not only increases the p53 acetylation but also induce apoptosis in monocytic THP-1 cells. These data actually place javamide-II in a potentially complex/indistinct position in elucidating the mechanism how the amide may have overall effects on Alzheimer's disease, cancer and others. In fact, p53 is a potent transcription factor involved in cell cycle arrest and apoptosis [32]. Due to its significance, the regulation of the protein is tightly carried out in the cells through multiple pathways/mechanisms, one of them is through the acetylation where Sirt enzymes are involved [32]. Our and other data showed that the inhibition of Sirt increases p53 acetylation which could accelerate cell death process [45]. Consequently, there is possibility that this may offset/alter potential benefits of javamide-II on Alzheimer's disease. For answering this question, we are currently preparing to investigate potential effects of the amide on Alzheimer's disease using animal models including Sirt2 knockout mouse. The outcomes of this study may provide further information about potential health effects of javamide-II on Alzheimer's disease and other diseases. Related to alpha-tubulin, the acetylation of alpha-tubulin was not increased significantly in the NG108-15 cells treated with the amide as described in this paper. However, we could observe a small increase of the alpha-tubulin acetylation in other cell line (e.g., THP-1 cells) (Fig 11), although subject to further investigation. The study has been done so far using only two different cell lines and the data suggest that javamide-II may increase the acetylation of alpha-tubulin in a cell-specific way. Therefore, in the future study, potential effects of javamide-II on alpha-tubulin acetylation in several different cells should be investigated in order to answer the question. Since javamide-II is a natural compound, it may be superfluous to expect that javamide-II could exert great activity and selectivity related to Sirt inhibition. In docking models, javamide-II was bonded to Sirt2 enzyme (4RMJ) with three potential hydrogen bonds (F96, R97 and S263) with ΔE = -58.6 Kcal/mol and to Sirt1 enzyme (4I5I) with two potential hydrogen bonds (L443 and S274) with ΔE = -42.7 Kcal/mol. These data showed that potential binding energy was little lower in Sirt2 than Sirt1, potentially inhibiting Sirt2 stronger than Sirt1, which was also supported *in vitro* inhibition assays performed in this study (Fig 3). Related to the Sirt2 inhibition, the amide is also to inhibit the enzyme competitively with respect to peptide substrate as shown in Fig 4B. This indicates that javamide-II found in coffee may inhibit Sirt2 via binding to the site where the substrate binds rather than that of NAD^+^, which was also supported by some docking models where the amide was posed away from the putative pocket of NAD^+^ binding site. However, to address this binding-site question unambiguously, X-ray crystallography experiments are currently being planned to determine the binding site of javamide-II in Sirt2. This study eventually provides the answer about where javamide-II binds to Sirt2 enzymes. Nonetheless, this study suggests that the amide is in fact a relatively potent Sirt1/2 inhibitor able to increase the acetylation of both Sirt1 and Sirt2 substrates (e.g., histone H3, cortactin, tubulin, p53), although the amide can inhibit Sirt2 to some extent better than Sirt1 *in vitro* assays. Therefore, there is a great possibility that the amide could be used as a parent/lead molecule which could lead to yielding Sirt inhibitors with great efficacy and selectivity through structural optimization. Currently, in our laboratory, several analogues of javamide-II are being synthesized for investigating their activity and selectivity related to Sirt inhibition, which may help find greater Sirt inhibitors with better selectivity.

## Abbreviations

HPLC: High Performance Liquid Chromatography
NMR: Nuclear magnetic resonance
AD: Alzheimer disease
DMSO: dimethyl sulfoxide
DIC: 1,3-diisopropylcarbodiimide
RT: retention times
ANOVA: Analysis of variance
SD: standard deviation
